# Affiliation to a Social Group as a Preventive Factor in Suicidal Behaviors in Children and Adolescents during the COVID-19 Pandemic

**DOI:** 10.3390/children10020333

**Published:** 2023-02-09

**Authors:** Jagoda Grzejszczak, Dominik Strzelecki, Agata Gabryelska, Magdalena Kotlicka-Antczak

**Affiliations:** 1 Department of Child and Adolescent Psychiatry, Medical University of Lodz, 92-216 Lodz, Poland; 2 Department of Affective and Psychotic Disorders, Medical University of Lodz, 92-216 Lodz, Poland; 3 Department of Sleep Medicine and Metabolic Disorders, Medical University of Lodz, 92-215 Lodz, Poland

**Keywords:** suicidal behaviors, suicide of children and adolescents, community protection, social group affiliation, COVID-19 pandemic, suicide prevention

## Abstract

Suicide is one of the most common causes of death in the population of children and adolescents. Available data show the continuous growth of this phenomenon and the ineffectiveness of prevention programs. Additionally, the COVID-19 pandemic significantly affected young people’s mental health, including an increased risk of suicidal behaviors due to limited direct contact with the school and peer groups in favor of the home environment. Therefore, the aim of this narrative review was to consider the risk factors and protective factors for suicidal behavior in the under-18 population, with a particular focus on the importance of belonging to a social group and building identification with it as a phenomenon protecting against suicidal behavior. Additionally, in this review, we evaluate how the COVID-19 pandemic affected these relationships. The PubMed database was used in the search with the following keywords: suicide, suicide behaviors, child and adolescent suicide behaviors, group affiliation, family affiliation, ethnicity, religious affiliation, and the COVID-19 pandemic, with articles published between 2002 and 2022 analyzed. Research conducted to date indicates that both sustained and stable family and peer relationships, as well as a sense of identification and belonging, noticeably reduce the risk of suicidal behavior. Ethnic or cultural affiliation seems to have been particularly important during the isolation in the home environment caused by the COVID-19 pandemic. Additionally, it has been shown that while in lockdown, contact through social media with individuals’ identification groups was associated with a reduced chance of emotional crises. Furthermore, regardless of cultural background, attachment to a particular group correlates with enhanced psychiatric state of children and adolescents. Thus, available data highlight the need for building and maintaining affiliations with suitable groups as a protective factor against suicidal behaviors.

## 1. Introduction

Suicidal behaviors include suicidal thoughts (ST), suicide plans (SP), and suicide attempts (SA) [1]. The suicide rate among 10–19-year-olds is estimated to be ~4 per 100,000, with large variations according to sex, age, and country of residence [2]. Suicide is a vital problem among minors, as it is the second most common cause of death among adolescents and the eighth leading cause of death among children aged 5 to 11 years in the world population [3,4]. In the late adolescents (15–19 years) age group, suicide was indicated as the most common cause of death in the female sex and the third most frequent cause of death in young males [5]. In general, the rates are higher among boys than girls worldwide, though the death rates for girls exceed those for boys in Bangladesh, China, India, and Nepal [6,7]. Overall, there has been a decrease in adolescent suicide rates over recent decades [8]. However, increases are reported in Southeast Asia and South America over the same period [7]. In the under-25 population, up to four times as many people are described as having a so-called cluster of suicides [9]. Suicide clusters can occur at a greater number of episodes than expected at a specific location, including institutions (e.g., schools, universities, psychiatric units, and youth offender units). The phenomenon may also occur over a wider geographic area due to contact with peers exposed to suicidal behavior not only directly but also through social media [10]. 

## 2. Materials and Methods

In the preparation of the following article, the search was based on the PubMed database with the following keywords: suicide, suicidal behavior, suicidal behavior of children and adolescents, group affiliation, family affiliation, ethnicity, religious affiliation, and the COVID-19 pandemic, with an analysis of articles published in English between 2002 and 2022. The literature search was performed on 29 October 2022. Additional relevant articles were included from references of initially identified articles. 

## 3. Risk and Protective Factors for Suicide Behaviors

There are proven factors that increase the risk of suicidal behavior, as well as protective factors. Details are presented in Table 1 [9,10,11]. Suicidal behavior differs between sexes, age groups, geographic regions, and sociopolitical settings and is variably associated with different risk factors, suggesting etiological heterogeneity [12]. In contrast, group affiliation is simultaneously emphasized as an important protective factor [13,14]. There are also studies indicating that the use of social media is a source of group support—as a protective factor against suicide [15,16,17]. Nevertheless, suicide among children and teenagers is still a taboo subject [18]. Therefore, the problem continuously requires comprehensive institutional measures, especially in communities of ethnic or religious affiliation [19].

Cultural identity and related group membership as a protective factor for mental health has been studied in many contexts [20]. Devaluation from an outside group was shown to have a smaller effect while an individual has a strong sense of ethnic identity, thus affecting psychological well-being [21]. Individuals who are part of a group are also more likely to be helped in emergency situations [22]. Furthermore, a lack of a sense of belonging carries a higher risk of aggressive and violent behavior or addiction in the adolescent population [23]. In addition, higher levels of a sense of social belonging have been shown to reduce the risk of psychopathological symptoms (including depressive symptoms) in the under-18 population [24].

## 4. Suicide vs. Group Affiliation

Given the seriousness of the problem of suicide in children and adolescents, various forms of support are still being sought [25]. However, despite the ongoing research, it is still hard to pinpoint the optimal form of support [26,27,28]. Considered mainstream, with the greatest availability, prevention programs aim to reduce the phenomenon’s scale and offer individual therapies for young people who have been directly affected [20,21,29]. In addition to individual interactions of various therapeutic trends, the effectiveness of therapeutic groups has been shown [22,23,30]. Researchers from Virginia (USA) have shown a significant effect of support from parents, a group of friends, and a school institution on reducing the frequency of suicidal ideation. The 143 surveyed studied students presented with almost double the risk (odds ratio (OR) 1.89) of suicidal behavior in the absence of concomitant support from school and a group of close friends compared to the control group [31]. Regardless of the many forms that support groups operate in|, their effectiveness is scientifically proven [32].

### 4.1. Origin

Factors contributing to the rate of, risks for, or protection against suicide may differ for individuals from cultures with different values and health beliefs [33]. Although studies report conflicting rates, most of them still show overall higher suicidal rates among Caucasian youth than any other ethnic group (e.g., 11.9/100,000 for Caucasians and 9.0/100,000 for Latinos) [34]. Asian Pacific Islander Americans (APIA) were significantly less likely than Latinos to have disclosed their suicidal intent to someone within a month of their deaths (OR = 1.98). Moreover, studies show that APIA and Latino descendants were significantly (OR = 0.79 and OR = 0.8, respectively, while for the Black population, OR = 0.58) more likely to have experienced interpersonal problems that contributed to their deaths than Black youths [35]. Researchers from Stanford University who analyzed youth suicidal behavior between 1999 and 2009 concluded that Native Hawaiian/Pacific Islander, multiracial, and American Indian/Alaska Native adolescents reported a significantly higher risk for suicide behaviors compared to their Asian, Black, Hispanic, and White peers (OR: 1.58–2.69) [36]. A study conducted in Utrecht, which regarded the impact of ethnic minority affiliation on the incidence of ST, showed this phenomenon to be almost twice as prevalent among Turkish minority adolescents compared to native Dutch teenagers. The exact reason for this disparity was not pinpointed. Hypotheses included a sense of loneliness among peers of a different nationality, lack of family support, or low economic status [37].

The issue of suicide in the context of ethnicity was already dealt with by Émile Durkheim [38]. Despite many hypotheses, the reasons for the disparity in suicidal behavior in young people of different nationalities have not yet been established [39]. An interesting theory refers to the model of the family system functioning. The study conducted by an international team shows a markedly increased rate of SA (OR = 11.6) in young Indian women when emotionally aroused. The SA had a more impulsive character if other family members were present at home. This may be interpreted as a women’s attempt to manifest their low position in the structure of the family system [40].

### 4.2. Indigenous Children

The child population of the continent’s indigenous people, who originally lived and may continue to live in a particular country or region, is a group at increased risk for mental health problems [41]. A systematic review including data on people in North America and Australia showed that native children face discrimination, negatively affecting their mental well-being. Furthermore, the inconsistency with other ethnic groups results in mental health problems in about half of the genuine individuals [42]. 

Indigenous youth suicide represents one of several health disparities burdening local populations. Like many other of these disparities, it can be understood as an expression of societal, historical, cultural, and familial trauma [43]. It turns out that preventive strategies targeting the individual are ineffective in indigenous societies [44]. Therefore, a novel approach named “culture as a treatment” has been proposed. It emphasizes the significance of interconnectedness in healing alongside the revitalization of traditional values (e.g., balance, community, family, culture, meaningful roles, spirituality, etc.) to reclaim community wellness [45]. Indeed, it turns out that suicide rates among young Canadians are highly variable. Much lower suicide rates were recorded among young members of Aboriginal communities, where the rights of minorities, their self-governance, language, and land rights were taken care of, or Aboriginal traditions were cultivated. For example, those with lower language knowledge had more than six times the suicidal rate [46]. In the context of Canada’s indigenous people, mention should be made of the Residential School System (RSS). The RSS was a network of boarding schools for indigenous people, which amounted to cultural genocide [47]. The Indian RSS, with many survivors reporting subsequent abuse, set in motion a cycle of trauma that resulted in alcohol and drug abuse problems, feelings of hopelessness, dependency, isolation, low self-esteem, suicide behaviors, prostitution, gambling, homelessness, sexual abuse, and violence. Although the RSSs are now closed down, their students are still alive. Data collected on a population of 611 former RSS students indicate more than double the prevalence of ST in this population (OR = 2.08) and almost double the frequency of SA (OR = 1.72) compared to non-residential school attendees [48]. The oppressive system of cultural eradication has left a clear mark on the mental state of Canada’s indigenous pediatric population [47]. Australian data show a twelve times higher suicide rate for Aboriginal children aged 10–14 compared to Australians of the same age. Possible explanations for this disparity include limited use of mental health support, living in remote areas from metropolitan areas, or living away from the family home [49]. A study on a group of just under 6.000 Norwegian adolescents, of whom about 9.2% were Sami (the indigenous people living in Northern: Norway, Sweden, Finland, and Russia), provides different results showing no significant differences in the incidence of suicidal behavior between Sami adolescents and their Norwegian peers [43,44]. 

Although most of the studied groups of indigenous children have an increased risk of suicidal behavior, the exact reasons for this link still require further examination [42]. 

### 4.3. Religion 

A lot of research has been conducted on the impact of religious group membership in the context of suicide prevention. Results are inconsistent; however, most confirm the protective effect of religious affiliation [45,46].

Studies, however, mainly focus on the general population, and the topic has not been thoroughly analyzed in the pediatric population. Irrespective of gender, low general suicide rates were found in Islamic countries, except for young Islamic women who commit suicide more often than peer men [47,48]. Research from Buddhist countries shows high suicide rates in women, and countries with a high percentage of inhabitants who do not declare affiliation to any religion are characterized by high suicide rates in men [49,50]. In Catholic countries, an association between secularization and more frequent suicidal behavior has been found [51]. Low suicide rates are recorded in Catholic countries with a high proportion of religious inhabitants [52,53,54]. Montreal researchers have attempted to determine how a religious group is affected. Fifteen young people between the ages of 14 and 18 who had attempted suicide in the past two years were included in the study. It was found that members of religious groups were more likely to receive a suggestion from other group members about the need to seek help in professional mental health services. In addition, undertaking the SA was more frequent among individuals who decided to leave their religious communities compared to active members of such communities. Yet, due to the very small study group, the results should be interpreted with great caution. In contrast, Orthodox Jews were the least likely to engage in suicidal behavior (7% of the study population versus 20% prevalence of SA among atheists) [55].

A series of data collected on suicidal behavior in young Muslim Women in the Middle East shows that such behaviors are increasingly common, and the rates are higher than in the male population. For example, for the Iranian population aged 15–24, the suicide rate for women is 4.7/100,000 while the rate for men is 1.2/100,000. The reasons for these statistics are complex, emphasizing the prevalence of psychiatric disorders, lack of help from mental health specialists, the dominant male role in the family system, or the expectation of early marriage [53]. 

Although religion is reported to be protective against suicide, the empirical evidence is inconsistent, and no definite answer can be provided at the time [56].

### 4.4. Family and Peer Group

In many suicidal behavior prevention programs, a peer group appears to be an important support element [57]. A study on 8776 Chinese aged 11–17 shows that they were more likely to attempt suicide when they had a small number of close friends compared to when they had more than three supportive peers in their imminent circle (OR = 1.41) [58]. On the other hand, research conducted on a group of 125 Black teenagers in Kentucky (USA) shows that the family system plays an important supportive role, and the peer group is less significant [59]. In contrast, data collected from 27 suicide-orphaned children in Denmark and the Republic of Ireland areas highlight the considerable impact of a peer support group for this type of experience [60]. The influence of the family system on suicidal behavior in children (especially the presence of SI in parents in the past) is reflected in a US study of 238 students aged 8–15 with a history of SI over the past three years. Results emphasize an interaction effect: low-quality relationships with parents predicted earlier emergence of SI among those young individuals whose parents had no history of SI [61]. It can be noted that an important factor in stabilizing children’s MH is the environment, in case it is unstable or dangerous, which leads to, among other things, widely understood violence (peer, domestic, sexual, neglect) [62]. Thus, the occurrence or severity of violence should be put into perspective as a risk factor for SA. It has to be pointed out that adolescents’ membership in peer groups with increased antisocial behavior was associated with an increased risk of emotional problems in their members [63].

Analysis of data on prevention programs for suicidal behaviors shows that only half of them are associated with reduced suicidal behavior [64]. For this reason, forms of assistance for children with suicidal behaviors should be continuously improved.

### 4.5. Immigration

Labor migration is a challenge for the globalized world due to its long-term effects, such as forming transnational families [65]. According to the United Nations, the number of economic migrants has nearly doubled over the past two decades [66]. The most vulnerable are Left Behind Children (LBC) whose parents leave to work (or leave in search of a source of income) and are taken care of by their extended families. A pronounced negative impact of this phenomenon on the mental state of LBC is observed in many countries in the Americas and South Asia [65]. The restructuring of the family system for economic reasons may result in low school performance, drop-out from school, conflicts with teachers and peers, anxiety, low self-esteem, tendency to feel depressed, apathy, suicidal behaviors, and substance abuse [67]. An increasing number of studies have discussed suicidal behaviors among LBCs [68]. Chinese authors showed a statistically significant increase in the incidence of ST (OR = 1.26) in LBC compared to the control group [69]. A study from Singapore recently reviewed results on the effectiveness of social interventions in suicide prevention. Most of the included studies showed that the risk of suicide in the group receiving various forms of social support was more than twice lower than in the control group (risk ratio (RR) 0.48) [70]. A social group can be considered a form of therapeutic group. A study of a nearly 3000 Chinese LBC from poor rural areas has shown that improving social support and nurturing relationships with loved ones (including a generation of grandparents) can help reduce the risk of suicidal behaviors in this population [71]. 

## 5. Community as a Protective Factor

**Figure 1:**
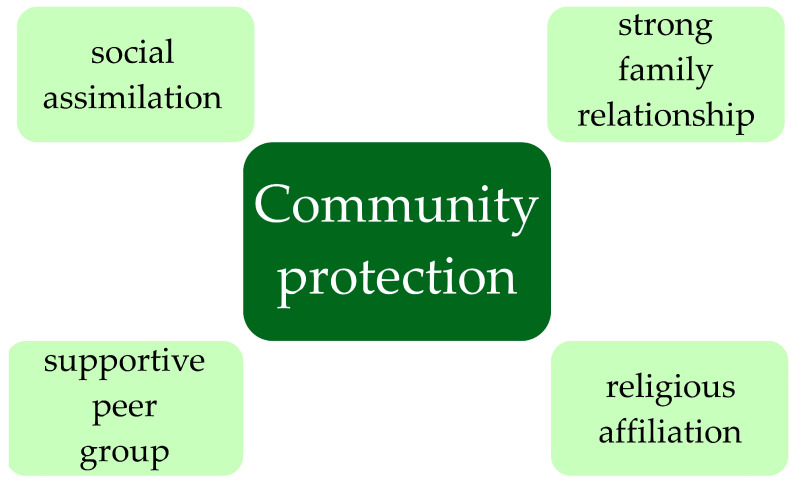
Form of community protection for suicide behaviors of youth.

Many empirical studies conducted on the general population show that being a member of a community reduces the risk of suicidal behaviors [72]. Detailed form of community protection for suicide behaviors of youth are presented in Figure 1. Social groups can have very different characteristics—ethnic, national, religious, or, finally, specific family systems and peer groups. Researchers point out that it is essential to enable the child to be brought up in a complete family, and the phenomenon of economic emigration increases the risk of psychological problems in LBC [65]. Some parents decide to emigrate with their children, which often generates difficulties in adapting to a community of foreign nationality. This can be associated with peer violence, low self-esteem, and suicidal behavior. The specific situation concerns indigenous children: in their populations, the risk of mental problems decreases as the quality of social assimilation increases [37]. Belonging to a religious group is not without significance. It has been proven that it is a protective factor in most religions, but the mechanisms of this dependence have not been fully understood. A sense of meaning, of belonging, and of order in the social structure seems likely to flow from religious affiliation [54]. The family system remains the basic protective factor, and many studies prove its superiority over the peer group. Close relationships, good communication, support, and parents’ acceptance significantly reduce mental problems, including suicidal behaviors [13].

## 6. Suicide Behaviors during the COVID-19 Pandemic

The COVID-19 pandemic greatly impacted the mental health of children and adolescents worldwide. In this period, an increase in depressive and anxiety symptoms, as well as a higher rate of suicidal behavior, has been observed [73]. The mechanism is complex, including both biological factors (effects of SARS-CoV2 infection) along with a wide variety of psychosocial factors, with social isolation and its adverse impact on the family system at the forefront [74]. Moreover, long-term effects can be expected due to the global economic crisis [75]. A meta-analysis from the University of Torino reported data on children under the age of 19 showed a 15% yearly increase in SA in 2021, a time of intense epidemiological restrictions [76]. Data from the survey conducted in Texas, US, collected from 9092 patients aged 11–21 years in a pediatric emergency department with a note of ST or SA, were compared with similar data collected before the pandemic period. The results indicated significantly higher rates of SI in March and July 2020 (1.60 times higher in March, 1.45 times higher in July) and higher rates of SA in February, March, April, and July 2020 (1.58, 2.34, 1.75, and 1.77 times higher, respectively, for each month) compared to the same months in 2019. The months with significantly higher rates of suicide-related behaviors appear to correspond with periods when COVID-19 stressors and community responses were heightened, indicating that youth also experienced increased stress during these periods [77]. According to data provided by the National Statistics Institute, 3941 people took their own lives in Spain in 2020, an increase of 7.4% over 2019. Currently, suicide is the leading cause of unnatural death in Poland in people aged 15–29 years. Of concern, suicide attempts in this age group increased by 250% in 2020 compared to previous years [78]. Japanese researchers attempted to collect data on SA in the under-20 population at the time of school closures during the COVID-19 pandemic. The data were compared with those obtained two years prior to the pandemic. They found no significant differences in SA taken during the school closure (incidence rate ratio (IRR) 1.15) compared to the pre-isolation period. However, the suicide rate increased in May (IRR = 1.34), when the school year ended, possibly suggesting that prolonged isolation was an important factor in increasing the risk of SA [79]. On the other hand, other Japanese research indicated no significant differences in suicide rates among children and adolescents during the first wave of the pandemic, but a significant increase in suicide rates (by as much as 49%) during the second wave of the pandemic (July to October 2020) was observed [80]. French researchers evaluated the incidence of hospitalization due to SA in 234 children and adolescents (mean age 13.4 years), showing a significant decrease in hospitalization due to SA (OR = 0.46). The reasons can be attributed not only to the limited availability of forms of assistance including hospitalization but also the lower impact of environmental factors such as violence at school, feeling of loneliness, and greater availability of adults in households [81]. The study from the US has shown that compared with the rate in 2019, a 31% increase in the proportion of mental health-related emergency department (ED) visits occurred among adolescents aged 12–17 years in 2020. The increase was much more pronounced in the female section of the study population [82]. Between 23 March and 10 May 2020, the National Society for the Prevention of Cruelty to Children Childline service delivered 30,868 counseling sessions to children and young people, 36% of which were about mental and emotional health concerns, and 13% were about ST. Samaritans also observed a 12% increase in calls made between 2 a.m. and 6 a.m. compared with previous years and longer phone sessions (40% longer conversations on average) [83].

Data from Korea on adolescents indicate that more than 35% of students in this group have experienced an emotional crisis, possibly leading to SA related to the conflict in the family system and insufficient support at home [84].

Researchers in Mexico are speaking out on the topic of protective factors. It is estimated that 2.8 million, or 10% of youth in Mexico, dropped out of school during the first six months of the pandemic. The study shows that attending online classes during the pandemic was associated with reduced odds of attempting suicide, as was reduced household spending (OR = 0.3) [85]. Both of these findings likely reflect the positive impact of enjoying a higher socioeconomic status: children from more economically comfortable families were not forced to abandon school, and their families were in a position to cut spending without major adverse consequences [86].

A meta-analysis by UK researchers on mental health in the era of the COVID pandemic in specific burden groups—healthcare workers, patients with comorbidities, and children and adolescents—provides a number of arguments regarding the positive impact of social aspects on mental health in effect reducing the risk of suicidal behavior. The data collected include peer support/having a cohesive team and social support in addition to protective factors such as previous experience or individual resilience [87]. 

The team from Taiwan and China, summarizing a 13-year prospective cohort study, also notes the importance of ongoing parental support and education as a key protective pillar in the era of the COVID pandemic [88].

Data on 522 children aged 8–12 years from three public primary schools in the southwestern region of Japan provide information on the beneficial effects of the presence of family members on the individuals’ mental state. This study was the first to show that loneliness may exacerbate the negative impact of the COVID-19 pandemic on Japanese children’s emotional and behavioral functioning. Furthermore, the authors suggested that spending time with mothers can be a particularly important protective factor for mitigating the negative impact of the pandemic on the emotional and behavioral functioning among children. Furthermore, for Japanese children, spending time with other family members, such as fathers, grandparents, and siblings may also mitigate the pandemic’s negative impact on emotional and behavioral functioning [89]. Quite interesting is the point of view of a parent. Ethiopian researchers have observed that among factors affecting parental stress, a very important role is played by strong and stable family relationships, which are also considered protective factors for the younger generation [90].

Another interesting study evaluated the impact of isolation on the mental health of sexual minority youth living in the rural US. It was shown that being able to interact online with people belonging to the same identity groups provided a sense of support, and acceptance, as well as potentially being an important source of information and positively affecting mental health by reducing the risk of depressive symptoms [91]. 

**Figure 2:**
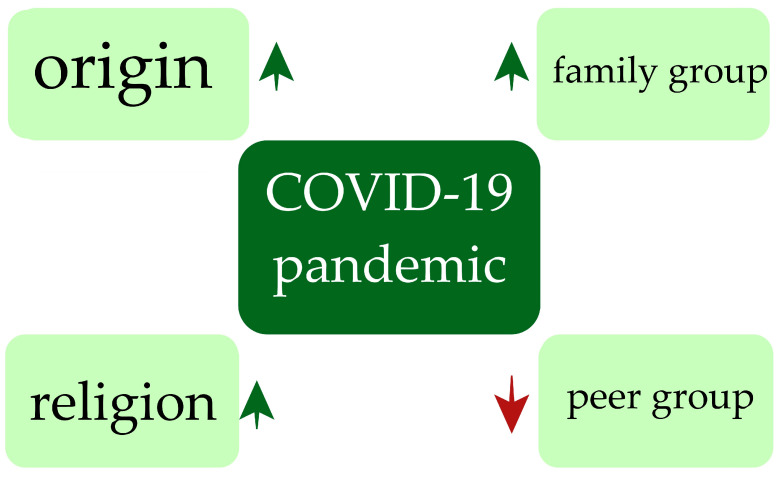
Effect of COVID-19 pandemic on availability of support through group affiliation. (↑—increase in support; ↓—decrease in support).

Number data of suicidal behaviors during the COVID-19 pandemic we presented in Table 2. Summary of effect of COVID-19 pandemic on availability of support through group affiliation we presented in Figure 2. 

It is necessary to continue collecting data on the prevalence of suicidal behavior in the child and adolescent population, especially during specific health and social challenges such as pandemics, which impose social isolation, as well as to develop effective prevention programs. It might be important to include in such programs the possibility of staying socially connected through online platforms or apps [92].

## 7. Conclusions

Young people’s sense of identity affects their mental health as young citizens [93]. Group affiliation including ethnicity affects a person’s psychological well-being [94]. In addition, social identity correlates with a greater accumulation of positive emotions [95]. Students with low levels of support from their social group are up to 6 times more likely to experience depressive symptoms [96]. This assumption was tested in the recent emergency in the era of the COVID-19 pandemic, which significantly affected the ability to contact affiliation groups as a source of support. The COVID-19 pandemic has disrupted the lives of people around the world. It is already apparent that several negative effects, especially regarding mental health, are affecting and will continue to affect the child and adolescent population in the future [97]. Mental health problems in youths during the pandemic seem to be a consequence of biological and psychosocial factors, with social isolation at the forefront [74]. They include not only depression and anxiety but also suicidal behavior [98]. Over the past three decades, there has been a marked increase in the incidence of both ST and SP or SA, even in children as young as a few years old [99]. Optimal forms of prevention of suicidal behavior in the youngest are still being sought [100]. This review highlighted the importance of adherence to social groups of different types as a preventive factor [101,102]. Among the protective factors of suicidal behavior, strong family ties, good communication, lasting peer bonds, but also religious affiliation are emphasized [11,90]. The time of the pandemic has significantly reduced the social contact of young people, moving them to an online environment [103]. They spent more time at home, often with their parents, whose work took a remote form [104]. The data collected so far on suicidal behavior in children and adolescents during the pandemic are inconclusive. Only some studies showed an increase in suicidal behavior interventions; some observed no significant change in the frequency of SA or the presence of SI, while some even reported a significantly reduced amount of crisis intervention [69,71,73]. The global social, economic, and emotional crisis caused by the COVID-19 pandemic has increased the risk of depression and anxiety in children and adolescents with suicidal behaviors as the most dramatic consequence, as evidenced by previous studies [87]. At the root of this phenomenon are economic problems in families, the limited availability of basic services (school, medical assistance), and the reduction of social contacts [88]. The increased stress to which parents are exposed can result in a deterioration of family relationships, including those between parent and child, which may result in a greater likelihood of conflict and risk of violence [55]. These cumulative difficulties potentially increase the risk of suicidal behaviors [88]. The specific conditions of everyday functioning (home isolation) sensitize us to the importance of the home environment and a sense of strong ties in the community of which the family is the primary example [89]. At the same time, however, driving family members’ time together at home can be an opportunity to strengthen bonds and thus re-educate symptoms of at least anxiety and positively affect the mental condition of the little ones [90]. It should also be mentioned that suicidal behavior is a complex problem and factors that are not directly related to the pandemic (personality traits, trauma, a loaded family history of suicide), although they are in close relation to the environment in which the young person is formed, i.e., family, cultural, or peer group can also increase the risk of suicide regardless of the circumstances of isolation [91]. Despite the ambiguous results regarding the influence of isolation on suicidal behavior, group affiliation is consistently mentioned as an important protective factor. Particular attention should be paid to the influence of ethnic or religious affiliation, which hypothetically may increase the effectiveness of suicide prevention in youths. This group relationship should be confirmed by conducting further studies. Regardless of the occurrence of a global crisis, prevention is important. The cooperation of different communities—psychologists, psychiatrists, internal medicine doctors, or educators—is crucial [105]. Helplines have long been practiced with good effectiveness in crisis situations among people under the age of 18 [106]. More recently, technology also seems promising—patient communication apps, provide an opportunity to directly contact a doctor in the event of mental deterioration including suicidal crisis [107].

## Figures and Tables

**Table 1:** Risk and protective factors for suicide behaviors in children and adolescents [10].

Risk Factors	Protective Factors
Non-Hispanic White Female sex Depressive disorder Anxiety disorder Being a victim of bullying Being uninsured Less guidance and supervision by parents Absence of a parental figure Having an unsafe home or school environment Family history of suicide Emotional traumas Other traumatic experiences Alcohol use disorder More time spent on the Internet Low income and socioeconomic status	Strong family relationships Family communication Parent’s role as a guardian in a child’s daily life Promote suicide prevention through the Internet Meaningful life Adequate nutrition (higher fruit intake and higher vegetable intake) reading books and watching movies Use the Internet for social purposes Faith or religiosity

**Table 2:** Number data of suicidal behaviors during the COVID-19 pandemic.

Country	Suicidal Behavior in the COVID-19 Pandemic	Reference
Italy (Torino)	Children under the age of 19 showed a 15% increase	[73]
US (Texas)	Higher rates of SA in February, March, April, and July 2020 (1.58, 2.34, 1.75, and 1.77 times higher, respectively, for each month) compared to the same months in 2019	[74]
Spain	People took their own lives in Spain in 2020, an increase of 7.4% over 2019	[75]
Japan	Significant increase in suicide rates (by as much as 49%) during the second wave of the pandemic (July to October 2020)	[77]
Korea	More than 35% of students in this group have experienced an emotional crisis, possibly leading to SA	[81]

## Data Availability

Not applicable.
